# Knee Bracing for Unicompartmental Osteoarthritis: A Service Evaluation

**DOI:** 10.1002/msc.70072

**Published:** 2025-03-05

**Authors:** Sarah Hetherington, Samantha Watson‐Smith, Felicity Evison, Caroline Miller

**Affiliations:** ^1^ Physiotherapy Integrated Musculoskeletal Services Solihull Community University Hospitals Birmingham NHS Foundation Trust Birmingham UK; ^2^ Health Data Science Team Research Development and Innovation Institute for Translational Medicine University Hospitals Birmingham NHS Foundation Trust Birmingham UK; ^3^ Physiotherapy Services Queen Elizabeth Hospital University Hospitals Birmingham Birmingham UK; ^4^ School of Inflection, Inflammation and Immunology, College of Medicine and Health University of Birmingham Birmingham UK

**Keywords:** bracing, knee osteoarthritis, physiotherapy, rehabilitation, service evaluation

## Abstract

**Objectives:**

This service evaluation examined the impact of offloader knee bracing for unicompartmental osteoarthritis (OA) on pain, quality of life (QoL) and activities of daily living (ADL).

**Design:**

The retrospective service evaluation was completed at two NHS community primary care clinics offering offloader knee bracing services. Between 2015 and 2023 patients prescribed offloader knee braces with unicompartmental osteoarthritis were invited to complete a Knee injury and Osteoarthritis Outcome Score (KOOS) at baseline, one, six and 12 months and at two and three years. KOOS data were analysed to assess the change in ADL, Pain and QoL domains from baseline to one, six and twelve months and up to three years using a paired *t*‐test. Demographic data collected included age, sex, and type of OA.

**Results:**

Two hundred and forty‐three patients were issued an offloader knee brace. There were statistically significant differences in pain and ADL for both braces at one and six months (*p* < 0.05). Participants issued with an ÖSSUR brace demonstrated statistically significant changes in pain and ADL for up to two years (*p* = 0.0101; *p* = 0.0153) and QoL up to one year (*p* = 0.0011). There was no statistically significant difference in either brace at three years. The ÖSSUR brace demonstrated a minimal clinically significant difference at one month for all domains, at one year for pain and ADL and two and three years for pain, ADL and QoL.

**Conclusions:**

Results indicate that offloader knee bracing for unicompartmental knee OA could reduce patients' pain, ADL and QoL in the long term.

## 1 Introduction

Osteoarthritis (OA) is a life changing degenerative joint disease which can have a significant impact on a person's physical and mental health (Yan et al. 2022; Shalhoub et al. 2022). OA is a major cause of pain and disability in the UK (Office for National Statistics 2017), with knee osteoarthritis documented as the most prevalent arthritic condition (Versus Arthritis 2024).

In the current economic climate, there is ever‐increasing pressure on the NHS to ensure utilisation of cost–effective treatments. Greater demand on orthopaedics because of an ageing population (Office for National Statistics 2017), increasing body mass index (BMI) (World Health Organization 2022) and increased patient expectations (Swarup et al. 2019) means consideration of alternative management strategies to surgery is imperative for knee osteoarthritis. Furthermore, there is a requirement to reduce the number of wear‐related revision surgeries by delaying Total Knee Replacement (TKR) surgery when possible (Weinsten et al. 2013).

BMI and knee muscle strength are key predictors in conserving knee joint health for knee osteoarthritis (Zheng and Chen 2015; Culvenor et al. 2016). For many patients, however, pain prevents them from being active and concomitantly being able to manage their bodyweight and strength around the affected joint. This can also have a negative impact on general wellbeing.

NICE guidelines (NG226) (2022) recommend ‘people with osteoarthritis who have biomechanical joint pain or instability should be considered for assessment for bracing/joint supports/insoles as an adjunct to their core treatments’. Indeed, it is suggested that knee bracing could be an effective alternative to patients for whom a TKR may not be a viable option or to reduce the level of disability for those awaiting surgery (Lee et al. 2017). A one year, randomised‐controlled trial concluded that there was ‘improvements in pain, function, and some aspects of quality of life’ when using a custom‐made knee brace for medial knee OA, including societal cost benefits (Gueugnon et al. 2021).

A TKR costs significantly more than knee bracing (Gandhi et al. 2023). The cost benefit of using braces may be even greater as this does not include any additional rehabilitation or costs associated with readmission because of complications such as infection or revision. It has also been demonstrated that patients who wore an offloader brace (*n* = 63) for two years or more did not require surgery at eight years and there was a similar QALY gain (mean increase in EQ5D of 0.435) compared with TKR at eight years follow‐up (Lee et al. 2017). Demonstration of these gains on a larger scale would be extremely advantageous.

There are many brands of offloader knee brace available. Knee braces work biomechanically by exerting a varus or valgus force across the affected joint away from the diseased compartment (Petersen et al. 2016). This action of offloading the forces through this area theoretically reduces pain for the wearer and concomitantly protects the joint from mechanical overload, thereby slowing the progression of disease (Schulz A, 2018).

A Cochrane review assessing the overall effect of the brace demonstrated limited high‐quality evidence in studies over 12 months and inconclusive advantages in short–term studies (Duivenoorden et al. 2015). Previous studies on the effects on knee bracing for unicompartmental OA suggest a positive short‐term reduction in pain levels and an increase in levels of activity (Divine and Hewett 2005; Haladik et al. 2014; Pagani et al. 2010); however, there is a dearth of evidence surrounding longer term outcomes of knee bracing for unicompartmental osteoarthritic knee.

The aim of this evaluation was to examine the impact of offloader knee bracing on unicompartmental OA in a primary care community musculoskeletal physiotherapy NHS setting, on pain quality of life (QoL) and activities of daily living (ADL) using the knee injury and osteoarthritis outcome score (KOOS) questionnaire.

## 2 Methods

A retrospective service evaluation was completed at two community primary care clinics in the West Midlands, United Kingdom. The service evaluation was registered with the Trust governance department (CARMS‐18905). These centres are the main providers of primary care knee bracing services to general practitioners in the area.

Patients prescribed an offloader knee brace for unicompartmental OA knee at the specialist knee clinic between July 2015 and January 2023 were included in the evaluation. Diagnosis of unicompartmental OA knee was confirmed with radiology within the previous 12 months. Patients with patellofemoral osteoarthritis in addition to unicompartmental osteoarthritis were included unless the patellofemoral pain was the main source of the pain. Patients who had tricompartmental OA or those who had received a cortico‐steroid injection within the previous 6 weeks, or with poor skin integrity were excluded from the evaluation.

**FIGURE 1:**
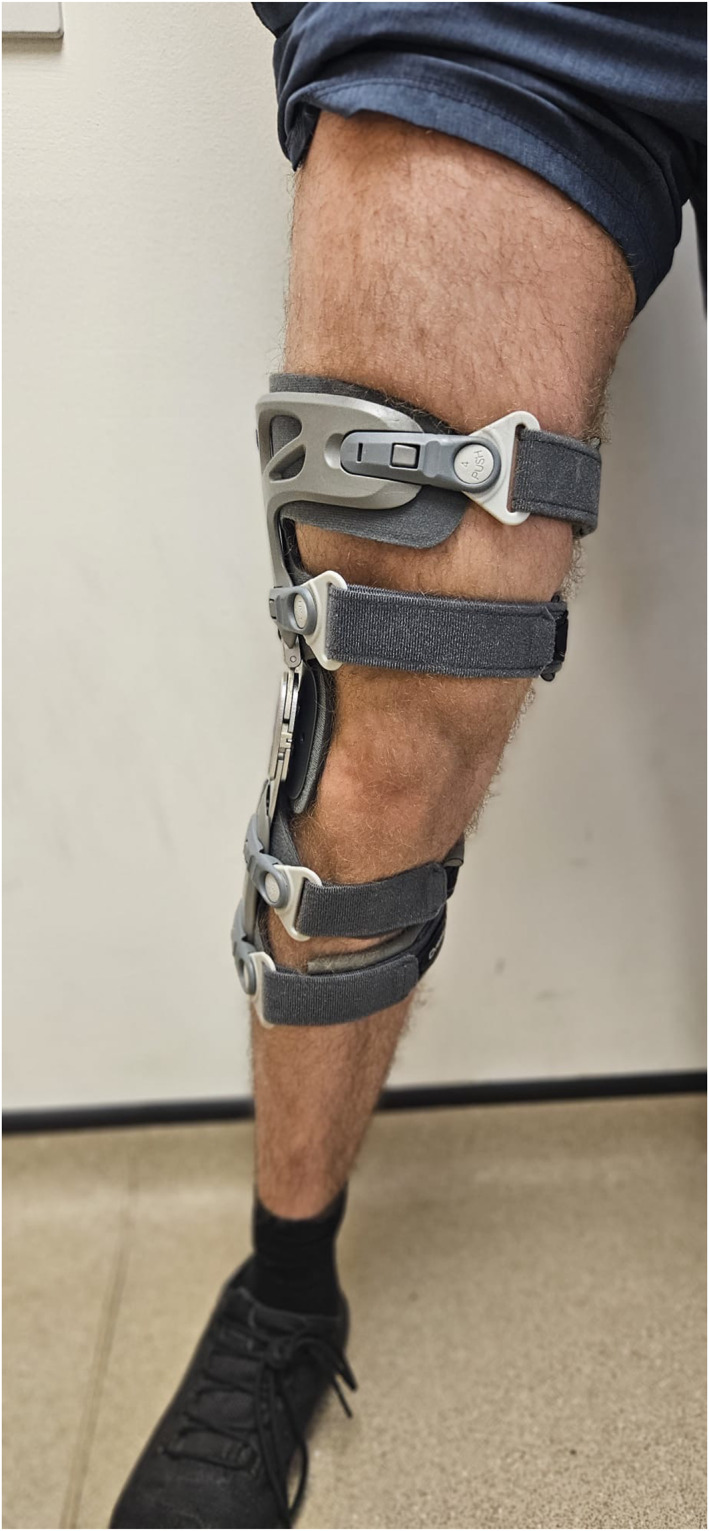
The Game Changer Universal OA Knee Brace.

**FIGURE 2:**
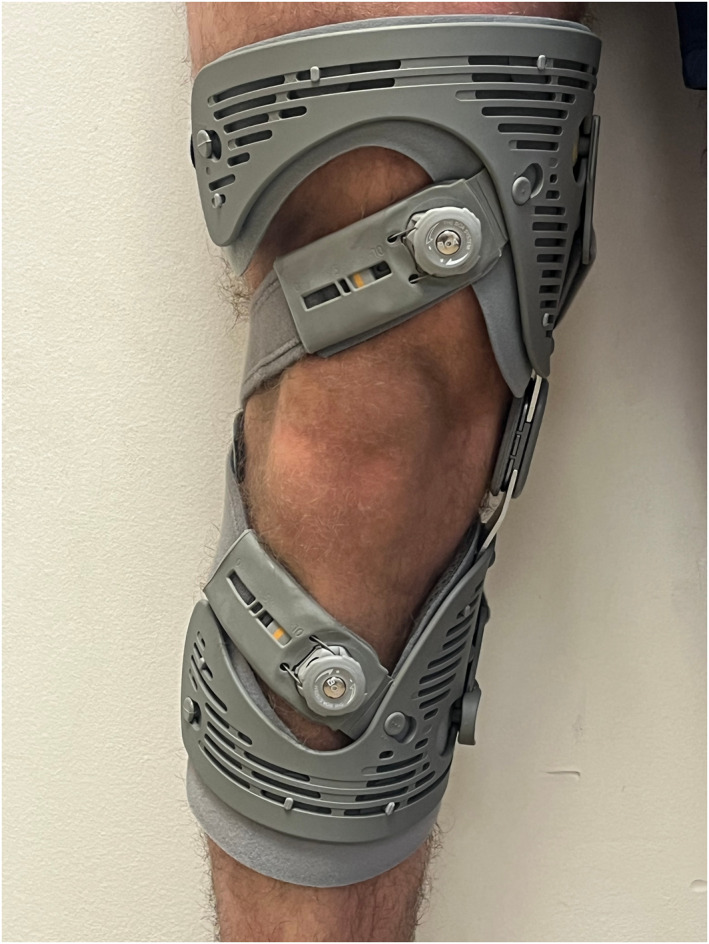
The ÖSSUR Unloader One Knee Brace.

The two braces used were a Game Changer (GC) Universal OA Knee Brace (Figure 1) and an ÖSSUR Unloader One Knee Brace (Figure 2) which are commissioned by the NHS Trust. Individual brace selection was determined by the severity of OA on radiology and patient leg morphology (e.g., size and shape). Patients were provided with a standardised advice and exercise sheet to minimise variation.

**FIGURE 3:**
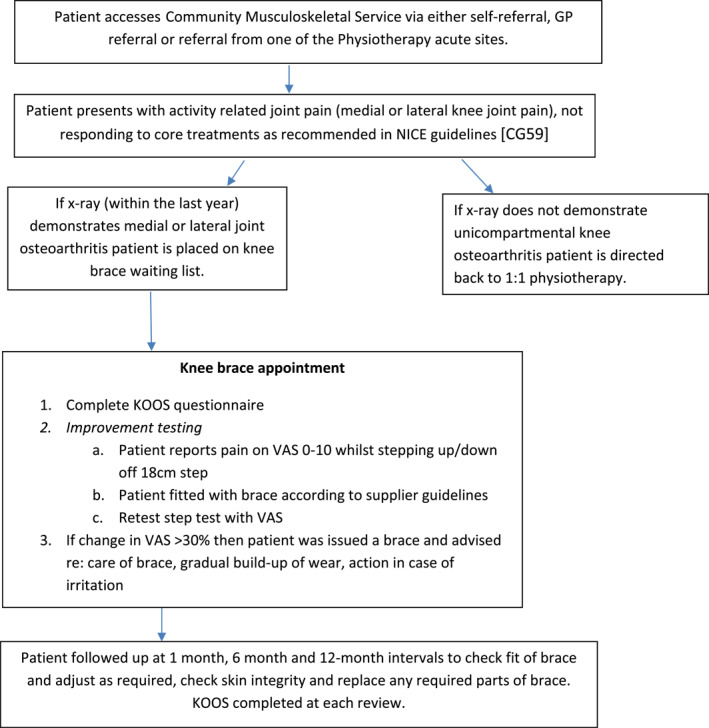
Treatment pathway for patients identified as potential knee brace candidates.

Each patient completed a Knee Injury and Osteoarthritis Outcome Score (KOOS) questionnaire at initial assessment, one month, six months, 12 months and annually thereafter. The KOOS has been shown to have ‘adequate internal consistency, test–retest reliability and construct validity’ in young and old adults with OA (Collins et al. 2016). Patients were contacted if they did not attend their follow‐up appointment to document reasons for non‐attendance. Figure 3 illustrates the pathway for patients potentially eligible for a knee brace.

Data from the KOOS questionnaire were extracted and inputted into Microsoft Excel. Patient notes were reviewed by S.H. and S.W.‐S. and demographics including medial/lateral compartment, brace type, age, sex, and clinic dates attended were extracted and inputted into the database. Data were analysed by an independent statistician (F.E.). Comparisons of demographics by brace type were undertaken using *t*‐test and chi‐squared tests. KOOS data were assessed for normality visually using QQ‐plots and histograms. The data were analysed to assess the change in KOOS (ADL, Pain and QoL domains) from baseline to one month, six months, twelve months and up to three years using a paired *t*‐test. Subgroup analyses were also undertaken based on age, sex and medial/lateral compartment OA. We focussed on these three chosen KOOS domains as these were agreed to be the most critical domains to measure in a recent knee and hip Core Outcome Set (Smith et al. 2019). Statistical analyses were undertaken in STATA SE 15.1 (StataCorp. 2017 *Stata Statistical Software: Release 15.1* College Station, TX: StataCorp LLC.). *p*‐values < 0.05 were considered statistically significant.

## 3 Results

Three hundred and eight patients attended the Knee Brace Clinic between July 2015 and January 2023. In total, 243 (79%) were issued with an offloader knee brace. Sixty‐five patients (19%) did not meet the improvement criteria (See Figure 1) and were not issued with a brace. Demographics of patients attending the clinic can be seen in Table 1. The ratio of males to females was relatively evenly split, with an average age of 61.5 years. The largest proportion of patients had medial compartment OA (93%). Slightly more ÖSSUR braces were issued (55.6%) compared to the Game Changer braces (44.4%).

**TABLE 1:** Details of patients attending knee bracing clinic.

	Issued knee brace	Not issued knee brace
Number	243	65
Age (mean ± SD)	61.5 (10.2)	65.1 (10.5)
Female	130 (53.5%)	36 (55.4%)
Male	113 (46.5%)	29 (44.6%)
Lateral	17 (7%)	7 (10.8%)
Medial	226 (93%)	56 (86.2%)
Other	0	2 (3.1%)
Game Changer	108 (44.4%)	18 (27.7%)
ÖSSUR	135 (55.6%)	47 (72.3%)

Demographics of patients issued with either Game Changer or ÖSSUR braces are presented in Table 2. There were no statistically significant differences between brace types. Slightly more women were issued a Game Changer brace (56.9%), and more men were issued an ÖSSUR brace (49%), as shown in Table 2.

**TABLE 2:** Demographics by brace type.

	Game Changer	ÖSSUR	*p*‐value
Number	108 (44.4%)	135 (55.6%)	
Age (mean ± SD)	62.5 (9.6)	60.1 (10.3)	0.0622
Female	66 (56.9%)	73 (51%)	0.328
Male	50 (43.1%)	70 (49%)	
Lateral	7 (6.5%)	7 (5.2%)	0.667
Medial	101 (93.5%)	128 (94.8%)	

There was a high attendance rate (> 75%) for patients allocated both the Game Changer and ÖSSUR braces at one month (See Table 3). However, by one year, attendance for patients with both braces had dropped. There were higher attendance rates for patients with the ÖSSUR brace.

**TABLE 3:** Clinic attendance rates per brace.

Follow up	Game Changer	ÖSSUR
1 month	89 (76.7%)	122 (85.3%)
6 months	45 (38.8%)	83 (58.0%)
1 year	28 (24.1%)	59 (41.3%)
2 years	9 (7.8%)	23 (16.1%)
3 years	4 (3.4%)	17 (11.9%)

Over the 3 years, there was a high number of dropouts (*n* = 170). Reasons for dropouts were categorised and are shown in Supporting Information S1.

### 3.1 Change in KOOS Domains Over 3 Years (Statistically Significant Changes)

Table 4 illustrates the statistical differences in each of the KOOS domains over a three‐year period whilst using a brace. There were statistically significant differences in pain and activities of daily living (ADL) for both braces at one and six months. There were no statistically significant differences in QoL.

**TABLE 4:** Mean KOOS domain scores with knee bracing over three years.

		Game Changer		ÖSSUR	
Time	KOOS domains	Mean (SD)	*p*‐value	Mean (SD)	*p*‐value
1 month	Pain	48.51 (2.12)	*p* < 0.0001	53.94 (1.58)	*p* < 0.0001
	ADL	50.46 (2.47)	*p* = 0.0001	58.70 (1.84)	*p* < 0.0001
	QOL	29.09 (2.05)	*p* = 0.1664	33.51 (1.82)	*p* < 0.0001
6 months	Pain	45.35 (2.83)	*p* = 0.0435	52.05 (2.46)	*p* = 0.0132
	ADL	51.09 (3.44)	*p* = 0.0169	57.54 (2.60)	*p* = 0.0088
	QOL	27.17 (2.54)	*p* = 0.3884	35.16 (2.73)	*p* = 0.0054
1 year	Pain	41.67 (4.34)	*p* = 0.4978	53.28 (2.67)	*p* = 0.0041
	ADL	42.79 (4.27)	*p* = 0.9047	59.31 (2.87)	*p* = 0.0043
	QOL	26.01 (4.21)	*p* = 0.8251	34.48 (2.82)	*p* = 0.0011
2 years	Pain	49.07 (7.71)	*p* = 0.0564	56.82 (2.97)	*p* = 0.0101
	ADL	54.90 (8.22)	*p* = 0.2598	62.81 (3.73)	*p* = 0.0153
	QOL	35.42 (5.62)	*p* = 0.1725	38.28 (4.16)	*p* = 0.0846
3 years	Pain	33.73 (11.62)	*p* = 0.8172	51.61 (4.85)	*p* = 0.1350
	ADL	39.71 (14.02)	*p* = 0.5496	56.89 (5.12)	*p* = 0.1928
	QOL	27.66 (10.28)	*p* = 0.3896	33.88 (4.83)	*p* = 0.1230

In the ÖSSUR, there are statistically significant differences in pain, ADL and QOL up to 6 months and then at 1 year pain and QoL and at 2 years pain and ADL. There were no statistically significant differences in either brace at 3 years.

### 3.2 The KOOS Minimal Clinically Important Difference

A minimal clinically important difference (MCID) of 8–10 has been suggested for the KOOS (Roos and Lohmander 2003). Table 5 illustrates the differences in the KOOS domains over the three years. The ÖSSUR brace demonstrated a MCID at one month for pain, ADL and QOL. At 1 year the ÖSSUR demonstrated a MCID for pain and ADL (but not statistically significant) and at 2‐ and 3‐year pain, ADL and QOL all show a clinically important change (but only pain and ADL at 2 years were statistically significant).

**TABLE 5:** Minimum clinical important difference KOOS.

Change		Game Changer Difference (SD)	ÖSSUR Difference (SD)
Baseline to 1 month	Pain	7.68 (1.58)	10.44 (1.39)
	ADL	5.88 (1.59)	10.07 (1.48)
	QOL	1.99 (2.07)	8.54 (1.53)
Baseline to 6 months	Pain	4.38 (2.29)	6.36 (2.32)
	ADL	5.10 (2.27)	6.40 (2.29)
	QOL	1.39 (2.30)	7.91 (2.71)
Baseline to 1 year	Pain	0.19 (2.83)	8.36 (2.50)
	ADL	−2.08 (3.47)	8.50 (2.69)
	QOL	−1.08 (3.68)	7.84 (2.33)
Baseline to 2 years	Pain	4.80 (6.03)	10.38 (4.09)
	ADL	3.21 (6.90)	10.29 (4.31)
	QOL	2.27 (4.24)	9.51 (5.58)
Baseline to 3 years	Pain	2.78 (10.54)	9.26 (4.07)
	ADL	9.31 (9.88)	8.09 (4.91)
	QOL	8.33 (7.16)	8.68 (5.28)

### 3.3 Comparisons Between Sexes

Although there appears to be no statistically significant difference with the Game Changer and the ÖSSUR braces in females at more than one month, there were both clinically and statistically significant changes for males in all three domains (Supporting Information S2). There were significant changes in QoL with the ÖSSUR for up to one year (8.87 [2.07] *p* < 0.001; 14.86 [3.59] *p* = 0.002; 11.04 [3.33] *p* = 0.005 at 1, 6 and 12 months, respectively). There were also significant changes for up to two years in Pain (12.58 [2.71]; *p* = 0.0448) and ADL (12.55 [5.50]; *p* = 0.0386) seen in males using the ÖSSUR. The Game Changer brace showed a statistically but not clinically significant difference in pain (6.35 [2.24]; *p* = 0.0042) and ADL (6.72 [2.37]; *p* = 0.0029) at one month in males.

### 3.4 Medial Cohort

For those with medial compartment OA, statistically and clinically significant differences were seen in pain (10.43 [1.45] *p* < 0.001; 6.78 [2.47] *p* = 0.0172; 8.31 [2.73] *p*‐0.0067; 10.98 [4.17] *p* = 0.0152 at 1, 6, 12 and 24 months, respectively) and ADL (10.37 [1.54] *p* < 0.001; 7.67 [2.37] *p* = 0.004; 9.11 [2.95] *p* = 0.0043; 10.8 [4.37] *p* = 0.0216) up to two years following provision of the ÖSSUR brace (Supporting Information S3). QoL was significant for ÖSSUR participants at one year (8.89 [2.45] *p* = 0.0011). The Game Changer brace showed statistically but not clinically significant differences up to six months in pain (4.54 [2.34] *p* = 0.0403) and ADL (5.20 [2.41] *p* = 0.0213).

### 3.5 Aged 60 and Under Cohort

For the 60 and under cohort, there was a very short‐term change in pain and ADL scores at one month for the Game Changer and ÖSSUR braces, and additionally for QoL with the ÖSSUR brace. In terms of clinically significant difference, ÖSSUR demonstrated positive results at pain and ADL at one month. Examining longer term results, the ÖSSUR group showed clinically (but not statistically) significant differences in pain, ADL and QOL at two years, and at three years a clinically (but not statistically) significant difference in QOL (Supporting Information S4).

### 3.6 Age 61 and Above

In the age 61 and above cohort, the Game Changer showed a statistically significant difference in pain (8.07 [2.14] *p* = 0.004) and ADL (6.73 [2.04] *p* = 0.0012) at one month only. For the ÖSSUR, both clinically and statistically significant differences were noted up to a year one year in pain (11.34 [1.86] *p* < 0.001; 10.33 [3.02] *p* = 0.0015; 10.94 [2.79] *p* = 0.0006, at 1, 6 and 12 months, respectively) and ADL (11.79 [2.15] *p* < 0.001; 10.48 [2.99] *p* = 0.0012; 10.41 [3.69] *p* = 0.0094) and up to six months for QoL (9.48 [2.09] *p* < 0.001; 9.29 [3.41] *p* = 0.0097).

## 4 Discussion

The aim of this service evaluation was to explore the effectiveness of using an offloader brace in adults with unicompartmental knee OA on pain, QoL and ADL. The evaluation analysed a large sample population of 243 patients over 3 years. Results of the overall cohort show a statistically significant reduction in pain and improvement in ADLs up to two years and a statistically significant improvement in QOL up to one year. Clinically significant differences, however, were found in all 3 categories ‐ pain, ADL and QOL at the 3‐year mark.

Our findings are consistent with those of a shorter‐term study by Ostrander et al. (2016), where patients were found to have significantly less pain and improved activity levels when examined up to 6 months. Similarly, Hjartarson and Toksvig‐Larsen (2018) found statistically significant improvements in pain and ADL up to 52 weeks. However, contrary to our findings, Hjartarson reported no statistically significant difference in QoL. Our study included a larger number of participants (*n* = 243) compared to Ostrander et al. 2016 (*n* = 31) and Hjartarson and Toksvig‐Larsen (2018) (*n* = 149), however, with a similar mean age (61, 63 and 59, respectively) in all three studies.

Comparing findings between the different sexes, in this evaluation, suggests that offloader bracing may be more effective in males up to 2 years. This is contrary to Lee et al.'s (2017) findings, who concluded ‘no significant difference in outcome’ regarding gender when evaluating effectiveness using the EQ‐5D‐3 L. The use of a different outcome measure may account for the differences; however, the use of a region‐specific patient‐reported outcome measure (the KOOS) is important to identify joint related patient reported changes. The current cohort has a similar gender distribution to Lee et al's 2017 study; however, the mean age of participants in the current study is 61 years compared to 51 in Lee et al's study. The older cohort in our study may have indicated that adults had more advanced osteoarthritis and therefore would potentially make larger changes in the KOOS specific domains. Overall, the number of participants in this evaluation greater than those included in Lee et al's (2017) study hence perhaps providing more confidence in these results.

## 5 Strengths and Limitations

This was a service evaluation with a very long follow‐up with data collected up to three years post provision of an offloader knee brace. We used an independent statistician to reduce bias. Two specialist physiotherapists ran the clinic, both with over 8 years of experience specifically in knee bracing. Decision making regarding the type of brace selected for the patient may vary depending on clinician bias towards brace preference. Brace selection was determined depending on the severity of OA on radiology and response in clinic to pain and fit.

As this was a service evaluation, patients were not restricted from using other treatment modalities which may have impacted results. Finally, there was a large drop out of patients at both the two‐ and three‐year assessment periods. This has impacted the validity of the results and needs to be considered when interpreting these data points. However, it does compare with other long‐term studies (Ostrander et al. 2016; Hjartarson and Toksvig‐Larsen (2018)). It may be that patients who were doing well with braces did not attend. The authors (SWS and SH) made all efforts to contact patients if they did not attend and recorded reasons for nonattendance.

## 6 Conclusion

The results of our service evaluation reflect our clinical experience in that offloader knee bracing significantly improves pain and ADL up to two years and QoL up to one year, with clinically important differences in pain, ADL and QoL at 3 years in a community MSK NHS physiotherapy setting. Further research should focus on whether there is any difference in provision of offloader bracing by age or gender.

## Author Contributions

**Sarah Hetherington:** conceptualisation, methodology, investigation, data collection, project administration, writing – original draft, writing – review and editing. **Samantha Watson‐Smith:** conceptualisation, methodology, investigation, data collection, project administration, writing – original draft, writing – review and editing. **Felicity Evison:** formal statistical analysis, visualisation, writing – review and editing. **Caroline Miller:** project management, project administration and co‐ordination, writing – original draft, writing – review and editing.

## Conflicts of Interest

The authors declare no conflicts of interest.

## Supporting information

Supporting Information S1

Supporting Information S2

Supporting Information S3

Supporting Information S4

## Data Availability

The data that support the findings of this study are available from the corresponding author upon reasonable request.
