# Supplementary material for: Knee Bracing for Unicompartmental Osteoarthritis: A Service Evaluation
Source: Musculoskeletal Care. 2025 Mar 5;23(1):e70072. doi: 10.1002/msc.70072 (PMC11882409; doi:10.1002/msc.70072)
Supplement: Supplementary file 1 — Supporting Information S1 [file MSC-23-e70072-s003.docx]

**Appendix 1 - Drop out reasons/ categories**

| A | Knee replacement surgery/ referred for orthopaedic opinion/ stem cell treatment/ arthroscopic chondroplasty | 14 |
| --- | --- | --- |
| B | Didn’t feel brace was effective anymore | 13 |
| C | Symptoms improved so stopped using brace | 15 |
| D | Slipping/ poor fit of brace | 4 |
| E | Skin irritation | 2 |
| F | Deceased | 2 |
| G | Change in lifestyle/ retired | 2 |
| H | Moved out of area/ unable to contact | 113 |
| I | Had corticosteroid injection | 5 |
