# Supplementary material for: Knee Bracing for Unicompartmental Osteoarthritis: A Service Evaluation
Source: Musculoskeletal Care. 2025 Mar 5;23(1):e70072. doi: 10.1002/msc.70072 (PMC11882409; doi:10.1002/msc.70072)
Supplement: Supplementary file 2 — Supporting Information S2 [file MSC-23-e70072-s004.docx]

**Supplementary File 2 – Male and Female Data**

|  |  |  |  |  |  |  |  |  |  |  |  |  |
| --- | --- | --- | --- | --- | --- | --- | --- | --- | --- | --- | --- | --- |

**Female cohort –** **Mean KOS domain scores with knee bracing**

|  |  | Gamechanger | | Ossur | |
| --- | --- | --- | --- | --- | --- |
| **1 month** | **Pain** | **45.46 (2.78)** | **p=0.0002** | **49.52 (2.17)** | **p<0.0001** |
|  | **ADL** | **45.62 (3.18)** | **p=0.0133** | **54.77 (2.52)** | **p<0.0001** |
|  | **QOL** | 26.15 (2.61) | p=0.0925 | **32.11 (2.72)** | **p=0.0007** |
| **6 months** | **Pain** | 44.15 (3.49) | p=0.1949 | 45.70 (2.98) | p=0.6766 |
|  | **ADL** | 50.84 (4.20) | p=0.1075 | 52.17 (3.46) | p=0.3341 |
|  | **QOL** | 27.68 (3.33) | p=0.0628 | 29.91 (3.70) | p=0.5214 |
| **1 year** | **Pain** | 38.12 (5.37) | p=0.6854 | 50.27 (4.17) | p=0.2756 |
|  | **ADL** | 38.48 (4.54) | p=0.4494 | 55.49 (4.18) | p=0.2101 |
|  | **QOL** | 20.83 (5.91) | p=0.8999 | 32.93 (4.68) | p=0.1000 |
| **2 years** | **Pain** | 50.40 (8.65) | p=0.3322 | 56.17 (3.73) | p=0.1217 |
|  | **ADL** | 57.35 (8.65) | p=0.6321 | 64.05 (4.79) | p=0.2478 |
|  | **QOL** | 39.29 (7.05) | p=0.6822 | 44.44 (5.65) | p=0.2767 |
| **3 years** | **Pain** |  |  | 44.10 (4.99) | p=0.9317 |
|  | **ADL** |  |  | 49.44 (5.29) | p=0.7746 |
|  | **QOL** |  |  | 30.47 (7.03) | p=0.7029 |

Female Cohort – Minimum Clinical Important Difference with KOOS

| **Change from Baseline** |  | **Gamechanger** | **Ossur** |
| --- | --- | --- | --- |
| **To 1 month** | **Pain** | 8.61 (2.19) | 8.09 (1.73) |
|  | **ADL** | 5.29 (2.15) | 9.48 (1.90) |
|  | **QOL** | 3.63 (2.58) | 8.19 (2.29) |
| **To 6 months** | **Pain** | 4.37 (3.28) | 1.19 (2.83) |
|  | **ADL** | 5.25 (3.15) | 2.84 (2.90) |
|  | **QOL** | 5.36 (2.76) | 2.08 (3.22) |
| **To 1 year** | **Pain** | -1.39 (3.37) | 4.65 (4.17) |
|  | **ADL** | -3.35 (4.33) | 5.32 (4.13) |
|  | **QOL** | -0.69 (5.44) | 5.77 (3.38) |
| **To 2 years** | **Pain** | 7.14 (6.77) | 8.95 (5.17) |
|  | **ADL** | 5.04 (10.0) | 8.33 (6.69) |
|  | **QOL** | 2.68 (6.23) | 9.03 (7.73) |
| **To 3 years** | **Pain** | 13.89 (25.0) | 0.35 (3.91) |
|  | **ADL** | 10.29 (20.59) | -0.84 (6.18) |
|  | **QOL** | 18.75 (18.75) | 2.34 (5.90) |

**Male cohort** - **Mean KOS domain scores with knee bracing**

|  |  | Gamechanger | | Ossur | |
| --- | --- | --- | --- | --- | --- |
| **1 month** | **Pain** | 52.78 (3.19) | **p=0.0042** | **58.15 (2.17)** | **p<0.0001** |
|  | **ADL** | 57.23 (3.66) | **p=0.0029** | **62.44 (2.61)** | **p<0.0001** |
|  | **QOL** | 33.21 (3.20) | p=0.7749 | **34.84 (2.43)** | **p<0.0001** |
| **6 months** | **Pain** | 47.22 (4.86) | p=0.0830 | **59.06 (3.70)** | **p=0.0045** |
|  | **ADL** | 51.47 (6.06) | p=0.0709 | **63.47 (3.74)** | **p=0.0090** |
|  | **QOL** | 26.39 (4.03) | p=0.4403 | **40.95 (3.88)** | **p=0.0023** |
| **1 year** | **Pain** | 46.58 (7.25) | p=0.1995 | **55.73 (3.45)** | **p=0.0043** |
|  | **ADL** | 48.76 (7.97) | p=0.5545 | **62.41 (3.90)** | **p=0.0085** |
|  | **QOL** | 33.17 (5.45) | p=0.5242 | **35.74 (3.46)** | **p=0.0046** |
| **2 years** | **Pain** | 47.22 (15.29) | p=0.0793 | **57.21 (4.29)** | **p=0.0448** |
|  | **ADL** | 51.47 (16.85) | p=0.1625 | **62.06 (5.34)** | **p=0.0386** |
|  | **QOL** | 30 (9.56) | p=0.1045 | 34.58 (5.65) | p=0.1974 |
| **3 years** | **Pain** | 32.22 (11.87) | p=0.9114 | 57.17 (7.30) | p=0.1269 |
|  | **ADL** | 38.53 (15.16) | p=0.7030 | 62.30 (7.77) | p=0.1072 |
|  | **QOL** | 27.5 (11.96) | p=0.7003 | 36.36 (6.78) | p=0.1325 |

**Male Cohort – Minimum Clinical Important Difference with KOOS**

| **Change from Baseline** |  | **Gamechanger** | **Ossur** |
| --- | --- | --- | --- |
| **To 1 month** | **Pain** | 6.35 (2.24) | 12.63 (2.12) |
|  | **ADL** | 6.72 (2.37) | 10.63 (2.26) |
|  | **QOL** | -0.36 (3.43) | 8.87 (2.07) |
| **To 6 months** | **Pain** | 4.41 (2.89) | 12.84 (3.59) |
|  | **ADL** | 4.84 (3.11) | 11.13 (3.53) |
|  | **QOL** | -5.15 (3.60) | 14.86 (4.29) |
| **To 1 year** | **Pain** | 5.56 (5.17) | 11.30 (3.03) |
|  | **ADL** | 1.23 (5.93) | 11.23 (3.72) |
|  | **QOL** | 0.52 (4.03) | 11.04 (3.33) |
| **To 2 years** | **Pain** | 14.81 (7.91) | 12.58 (5.71) |
|  | **ADL** | 5.88 (5.30) | 12.55 (5.50) |
|  | **QOL** | 6.25 (3.61) | 10.0 (7.39) |
| **To 3 years** | **Pain** | -13.89 (9.00) | 15.74 (6.48) |
|  | **ADL** | -5.88 (13.70) | 15.69 (7.29) |
|  | **QOL** | -6.25 (5.71) | 16.67 (8.53) |
