# Supplementary material for: Knee Bracing for Unicompartmental Osteoarthritis: A Service Evaluation
Source: Musculoskeletal Care. 2025 Mar 5;23(1):e70072. doi: 10.1002/msc.70072 (PMC11882409; doi:10.1002/msc.70072)
Supplement: Supplementary file 3 — Supporting Information S3 [file MSC-23-e70072-s002.docx]

**Supplementary File 3 - Medial OA**

**Medial cohort -** **Mean KOOS domain scores with knee bracing**

|  |  | Gamechanger | | Ossur | |
| --- | --- | --- | --- | --- | --- |
| **1 month** | **Pain** | 48.67 (2.28) | **p<0.0001** | 53.88 (1.64) | **p<0.0001** |
|  | **ADL** | 50.99 (2.62) | **p=0.0001** | 58.82 (1.89) | **p<0.0001** |
|  | **QOL** | 29.30 (2.17) | p=0.1670 | 33.35 (1.87) | **p<0.0001** |
| **6 months** | **Pain** | 45.24 (3.06) | **p=0.0403** | 52.48 (2.59) | **p=0.0172** |
|  | **ADL** | 51.51 (3.70) | **p=0.0213** | 58.76 (2.68) | **p=0.0040** |
|  | **QOL** | 27.98 (2.70) | p=0.2134 | 35.92 (2.87) | **p=0.0050** |
| **1 year** | **Pain** | 39.94 (4.47) | p=0.7583 | 53.42 (2.82) | **p=0.0067** |
|  | **ADL** | 41.48 (4.45) | p=0.7128 | 60.19 (2.99) | **p=0.0043** |
|  | **QOL** | 24.78 (4.40) | p=0.9548 | 35.07 (2.95) | **p=0.0011** |
| **2 years** | **Pain** | 49.07 (7.71) | p=0.0564 | 56.63 (3.09) | **p=0.0152** |
|  | **ADL** | 54.90 (8.22) | p=0.2598 | 63.0 (3.89) | **p=0.0216** |
|  | **QOL** | 35.42 (5.62) | p=0.1725 | 38.57 (4.33) | p=0.0756 |
| **3 years** | **Pain** | 33.73(11.62) | p=0.8172 | 51.80 (5.43) | p=0.1824 |
|  | **ADL** | 39.71(14.02) | p=0.5496 | 57.09 (5.71) | p=0.2569 |
|  | **QOL** | 27.68(10.28) | p=0.3896 | 33.46 (5.38) | p=0.1571 |

**Medial OA cohort – Minimum Clinical Important Difference with KOOS**

| **Change from baseline** | |  | **Gamechanger** | **Ossur** |
| --- | --- | --- | --- | --- |
| **To 1 month** | **Pain** | | 8.37 (1.62) | 10.43 (1.45) |
|  | **ADL** | | 6.13 (1.63) | 10.37 (1.54) |
|  | **QOL** | | 1.84 (2.02) | 8.22 (1.55) |
| **To 6 months** | **Pain** | | 4.54 (2.34) | 6.78 (2.47) |
|  | **ADL** | | 5.20 (2.41) | 7.67 (2.37) |
|  | **QOL** | | 2.29 (2.31) | 8.61 (2.87) |
| **To 1 year** | **Pain** | | 0.20 (2.79) | 8.31 (2.73) |
|  | **ADL** | | -2.57 (3.61) | 9.11 (2.95) |
|  | **QOL** | | -1.34 (3.73) | 8.89 (2.45) |
| **To 2 years** | **Pain** | | 9.44 (5.19) | 10.98 (4.17) |
|  | **ADL** | | 5.29 (6.97) | 10.81 (4.37) |
|  | **QOL** | | 3.75 (4.39) | 10.33 (5.54) |
| **To 3 years** | **Pain** | | -4.63 (10.41) | 8.52 (4.81) |
|  | **ADL** | | -0.49 (10.72) | 7.16 (5.82) |
|  | **QOL** | | 2.08 (8.01) | 10.42 (6.18) |
