# Supplementary material for: Knee Bracing for Unicompartmental Osteoarthritis: A Service Evaluation
Source: Musculoskeletal Care. 2025 Mar 5;23(1):e70072. doi: 10.1002/msc.70072 (PMC11882409; doi:10.1002/msc.70072)
Supplement: Supplementary file 4 — Supporting Information S4 [file MSC-23-e70072-s001.docx]

**Supplementary File 4 – Analysis by age**

**Aged 60 and under cohort (n=112) -** **Mean KOOS domain scores with knee bracing**

|  |  | Gamechanger | | Ossur | |
| --- | --- | --- | --- | --- | --- |
| **1 month** | **Pain** | **40.24 (3.55)** | **p=0.0027** | **51.41 (2.21)** | **p<0.0001** |
|  | **ADL** | **42.25 (3.83)** | **p=0.0345** | **57.40 (2.85)** | **p=0.0001** |
|  | **QOL** | 21.97 (2.91) | p=0.8363 | **29.82 (2.54)** | **p=0.0008** |
| **6 months** | **Pain** | 41.29 (4.02) | p=0.0981 | 47.56 (3.47) | p=0.5861 |
|  | **ADL** | 45.32 (4.87) | p=0.0661 | 54.91 (3.77) | p=0.5279 |
|  | **QOL** | 23.86 (3.60) | p=1 | 31.25 (3.94) | p=0.1439 |
| **1 year** | **Pain** | 35.46 (5.01) | p=0.5292 | 52.02 (3.91) | p=0.1725 |
|  | **ADL** | 36.25 (4.93) | p=0.7842 | 58.56 (4.12) | p=0.1047 |
|  | **QOL** | 17.28 (4.44) | p=0.4401 | 30.68 (3.98) | p=0.066 |
| **2 years** | **Pain** | 45.63 (9.88) | p=0.0772 | 58.97 (5.20) | p=0.1343 |
|  | **ADL** | 51.68 (11.28) | p=0.161 | 67.53 (6.44) | p=0.0634 |
|  | **QOL** | 33.93 (8.17) | p=0.1177 | 35.10 (7.24) | p=0.1437 |
| **3 years** | **Pain** | 28.89 (14.82) | p=0.534 | 50.00 (7.64) | p=0.4146 |
|  | **ADL** | 31.76 (17.29) | p=0.5695 | 56.68 (8.04) | p=0.4056 |
|  | **QOL** | 21.25 (13.05) | p=0.2835 | 26.70 (6.95) | p=0.333 |

**Age 60 and under cohort (n=112)** - **Minimum Clinical Important Difference with KOOS**

| **Change from baseline** |  | **Gamechanger** | **Ossur** |
| --- | --- | --- | --- |
| **To 1 month** | **Pain** | 7.07 (2.33) | 9.48 (2.08) |
|  | **ADL** | 4.55 (2.55) | 8.24 (2.02) |
|  | **QOL** | -1.70 (3.42) | 7.54 (2.26) |
| **to 6 months** | **Pain** | 5.03 (3.37) | 3.06 (3.50) |
|  | **ADL** | 4.27 (2.69) | 3.05 (3.41) |
|  | **QOL** | -1.49 (3.86) | 6.88 (4.25) |
| **to 1 year** | **Pain** | 1.39 (3.90) | 6.00 (4.00) |
|  | **ADL** | -3.40 (4.43) | 6.93 (4.05) |
|  | **QOL** | -5.86 (4.36) | 7.46 (3.61) |
| **to 2 years** | **Pain** | 12.22 (7.17) | 11.54 (7.18) |
|  | **ADL** | 6.47 (6.80) | 14.37 (7.02) |
|  | **QOL** | 5.00 (4.59) | 12.98 (8.30) |
| **to 3 years** | **Pain** | 0.69 (14.72) | 7.10 (6.00) |
|  | **ADL** | -2.57 (12.09) | 6.54 (6.98) |
|  | **QOL** | 6.25 (10.83) | 11.11 (9.07) |

**Age 61 and above cohort (n=131)-** **Mean KOOS domain scores with knee bracing**

|  |  | Gamechanger | | Ossur | |
| --- | --- | --- | --- | --- | --- |
| **1 month** | **Pain** | **53.87 (2.37)** | **p=0.0004** | **56.27 (2.22)** | **p<0.0001** |
|  | **ADL** | **55.77 (3.02)** | **p=0.0012** | **59.89 (2.39)** | **p<0.0001** |
|  | **QOL** | 33.70 (2.63) | p=0.0979 | **36.90 (2.54)** | **p<0.0001** |
| **6 months** | **Pain** | 49.07 (3.90) | p=0.2439 | **56.77 (3.36)** | **p=0.0015** |
|  | **ADL** | 56.37 (4.70) | p=0.1182 | **60.29 (3.58)** | **p=0.0012** |
|  | **QOL** | 30.21 (3.54) | p=0.1548 | **39.26 (3.72)** | **p=0.0097** |
| **1 year** | **Pain** | 49.21 (7.14) | p=0.7634 | **54.94 (3.47)** | **p=0.0006** |
|  | **ADL** | 50.74 (6.96) | p=0.912 | **60.29 (3.90)** | **p=0.0094** |
|  | **QOL** | 36.61 (6.74) | p=0.278 | 39.50 (3.75) | p=0.0024 |
| **2 years** | **Pain** | 53.89 (13.31) | p=0.4581 | 54.27 (2.17) | p=0.0023 |
|  | **ADL** | 59.41 (13.04) | p=0.769 | 57.22 (2.26) | p=0.1023 |
|  | **QOL** | 37.50 (8.15) | p=0.7717 | 42.05 (3.16) | p=0.4062 |
| **3 years** | **Pain** | 45.83 (20.83) | p=0.4365 | 53.82 (5.25) | p=0.1563 |
|  | **ADL** | 59.56 (24.26) | p=0.9188 | 57.17 (5.76) | p=0.335 |
|  | **QOL** | 43.75 (12.50) | p=0.7048 | 43.75 (4.87) | p=0.2037 |

**Age 61 and above cohort (n=112) -** **Minimum Clinical Important Difference with KOOS**

| **Change from baseline** |  | **Gamechanger** | **Ossur** |
| --- | --- | --- | --- |
| **To 1 month** | **Pain** | 8.07 (2.14) | 11.34 (1.86) |
|  | **ADL** | 6.73 (2.04) | 11.79 (2.15) |
|  | **QOL** | 4.33 (2.57) | 9.48 (2.09) |
| **To 6 months** | **Pain** | 3.82 (3.19) | 10.33 (3.02) |
|  | **ADL** | 5.82 (3.59) | 10.48 (2.99) |
|  | **QOL** | 3.91 (2.66) | 9.29 (3.41) |
| **To 1 year** | **Pain** | 1.39 (4.52) | 10.94 (2.79) |
|  | **ADL** | 0.63 (5.59) | 10.41 (3.69) |
|  | **QOL** | 6.25 (5.52) | 10.00 (2.95) |
| **To 2 years** | **Pain** | 6.67 (8.13) | 10.84 (2.67) |
|  | **ADL** | 4.12 (13.10) | 6.95 (3.87) |
|  | **QOL** | 2.50 (8.05) | 5.68 (6.55) |
| **To 3 years** | **Pain** | -15.28 (12.50) | 10.07 (6.34) |
|  | **ADL** | 3.68 (28.68) | 8.46 (8.17) |
|  | **QOL** | -6.25 (12.50) | 8.59 (6.13) |
